# Surveillance guidelines for disease elimination: A case study of canine rabies

**DOI:** 10.1016/j.cimid.2012.10.008

**Published:** 2013-05

**Authors:** Sunny E. Townsend, Tiziana Lembo, Sarah Cleaveland, François X. Meslin, Mary Elizabeth Miranda, Anak Agung Gde Putra, Daniel T. Haydon, Katie Hampson

**Affiliations:** a Boyd Orr Centre for Population and Ecosystem Health, Institute for Biodiversity, Animal Health and Comparative Medicine, College of Medical, Veterinary and Life Sciences, University of Glasgow, Glasgow, G12 8QQ, Scotland, UK; b Department of Control of Neglected Tropical Diseases, World Health Organization, 20 Avenue Appia, CH-1211 Geneva 27, Switzerland; c Global Alliance for Rabies Control, Humboldt St Suite One, Manhattan, KS, USA; d Disease Investigation Center Denpasar, Jalan Raya Sesetan 266, Denpasar 80223, Bali, Indonesia

**Keywords:** Zoonoses, Infectious disease, Epidemiological modelling, Dog rabies, Eradication

## Abstract

Surveillance is a critical component of disease control programmes but is often poorly resourced, particularly in developing countries lacking good infrastructure and especially for zoonoses which require combined veterinary and medical capacity and collaboration. Here we examine how successful control, and ultimately disease elimination, depends on effective surveillance. We estimated that detection probabilities of <0.1 are broadly typical of rabies surveillance in endemic countries and areas without a history of rabies. Using outbreak simulation techniques we investigated how the probability of detection affects outbreak spread, and outcomes of response strategies such as time to control an outbreak, probability of elimination, and the certainty of declaring freedom from disease. Assuming realistically poor surveillance (probability of detection <0.1), we show that proactive mass dog vaccination is much more effective at controlling rabies and no more costly than campaigns that vaccinate in response to case detection. Control through proactive vaccination followed by 2 years of continuous monitoring and vaccination should be sufficient to guarantee elimination from an isolated area not subject to repeat introductions. We recommend that rabies control programmes ought to be able to maintain surveillance levels that detect at least 5% (and ideally 10%) of all cases to improve their prospects of eliminating rabies, and this can be achieved through greater intersectoral collaboration. Our approach illustrates how surveillance is critical for the control and elimination of diseases such as canine rabies and can provide minimum surveillance requirements and technical guidance for elimination programmes under a broad-range of circumstances.

## 1 Introduction

Surveillance is a critical element in the control and elimination of infectious diseases [1]. Effective surveillance systems allow early detection and reporting of cases, vital for initiating timely responses and enabling informed decisions about when and where to intensify control efforts. Once interventions are implemented, surveillance is also essential to generate data on the progress and cost-effectiveness of such programmes, which are essential for their sustainable implementation. In practice the quality of surveillance and therefore the probability of disease detection can vary considerably, with consequences for disease control such as outbreak containment or discontinuation of control measures once freedom from disease has been achieved. Weak surveillance may therefore result in delayed control interventions and complacency [2] and can jeopardize chances of disease elimination [3]. As control efforts progress towards elimination, surveillance becomes even more critical in order to detect new incursions. “Unless an effective reporting and surveillance programme is developed, there is no prospect whatsoever for a successful eradication programme” D.A. Henderson [4].

Rabies is one of the most feared zoonoses, nearly always resulting in fatal acute encephalitis [5]. Although rabies is maintained and transmitted by a wide range of species and may never be eradicated from all species, it is feasible to eliminate canine rabies [6], which is responsible for the vast majority of human cases worldwide and is of the greatest public health concern [7,8]. Canine rabies is not only a major burden in endemic countries where thousands of human deaths are estimated to occur annually [7], but also in previously rabies-free areas where risks of re-emergence have been increasing over the last decade [e.g. 9, 10, 11]. A ‘One Health’ approach is the most effective way of protecting humans from canine rabies, as infection is maintained in domestic dog populations. A number of countries have achieved considerable successes in canine rabies elimination through mass dog vaccination [12–14]. The feasibility and cost-effectiveness of this approach has been strongly advocated in recent years [15], with major international public and animal health organisations declaring global canine rabies elimination as a realistic goal (e.g. WHO http://www.who.int/rabies/bmgf_who_project/en/index.html; OIE http://www.oie.int/en/for-the-media/editorials/detail/article/oies-commitment-to-fight-rabies-worldwide). The degree of success of national and global canine rabies elimination efforts is however heavily reliant on effective epidemiological surveillance, which should ensure that intervention impacts can be monitored through time and outbreak responses initiated where necessary. Indeed, response times to incursions are dependent on the speed of first detection (Table 1), hence surveillance plays a major role in triggering an early response.

For vaccine preventable diseases, surveillance typically improves once a control programme gets underway, as observed during eradication efforts for polio, and more generally during the expanded programme on immunization (EPI) for the control of measles and other childhood infections [1,16]. However, in developing countries routine surveillance may initially be vestigial to non-existent with limited reporting accounting for substantial underestimation of cases [3]. For example, prior to the establishment of intensive surveillance activities for smallpox, estimates of reporting rates in Indonesia and West Africa varied from <1% of cases to 8% in urban areas [Keja (1968) reported in 3, 17]. Underreporting is particularly severe for diseases of zoonotic origin where very few examples of well-integrated surveillance mechanisms exist [18]. For rabies, largely complete and well-functioning surveillance and data management systems are maintained in countries where canine rabies has either been eliminated (U.S.A. http://www.cdc.gov/rabies/location/usa/surveillance/index.html; Europe http://rbe.fli.bund.de (Rabies-Bulletin-Europe)) or is under control and prospects for elimination are good (South America http://siepi.panaftosa.org.br/anuais.aspx), in contrast to deficient surveillance operating in most endemic countries. Surveillance capacity is also often particularly weak in areas without a history of rabies. Health workers play an integral role in the surveillance of strictly human diseases, and in previously rabies free-areas the speed of response to outbreaks has sometimes depended on health workers identifying the disease in humans (e.g. Bali and Nias in Indonesia, Table 1). Ultimately a One Health approach involving the close cooperation of medical and veterinary workers is required for effective surveillance of rabies and other zoonoses [19].

Limited resources mean that trade-offs inevitably exist between maintaining sensitive surveillance systems and mobilizing responses to an incursion once detected by a less sensitive, passive surveillance system. Recent emergences of rabies highlight the risks posed, including massive economic repercussions and need for continuous public health and veterinary staff mobilization, from inadvertent introductions if effective surveillance and response measures are not in place [9,11,20]. In endemic areas surveillance for canine rabies typically involves passive reporting of clinically suspected human and/or animal cases, and ideally laboratory diagnosis of suspect animal cases particularly if they have caused possible human exposures (although in many areas this is not carried out). While a lack of proper diagnostic facilities often limits rabies surveillance, weak field capacity for investigating cases and poorly functioning reporting networks are perhaps a more enduring problem [18].

OIE guidelines for a ‘rabies free country’ in Article 8.10.2 of the Terrestrial Animal Health Code require “an effective system of disease surveillance is in operation” and “no case of indigenously acquired rabies infection has been confirmed in man or any animal species during the past 2 years.” [21]. WHO guidelines on the levels of surveillance needed to certify rabies-free status indicate that “a minimum number of samples from suspect cases” should be tested, and that “for domestic animals, in particular dogs and cats, the number of samples to be tested should be between 0.01–0.02% of the estimated population” [8]. However, clarity is still needed on for example, definitions for targeted surveillance, control strategies to rabies incursions and maintaining rabies-freedom, and quantitative surveillance assessments for decision-making within rabies elimination programmes.

Although surveillance is an essential component of control programmes, this is often not well recognized in developing countries and is exacerbated by poor infrastructure and health and veterinary capacity. However, it is precisely these countries where endemic canine rabies remains, and surveillance is therefore necessary to monitor the impact of any control efforts. In light of these surveillance issues, and growing advocacy for elimination of canine rabies, here we aim to understand how successful rabies control and elimination depends on the effectiveness of surveillance by investigating different containment strategies guided by surveillance indicators. Specifically we use the probability of disease detection to measure surveillance quality and ask how this affects the outcome of mass vaccination strategies, in terms of the extent of outbreak spread and time to as well as probability of elimination. We further investigate how probability of detection affects the certainty of declaring freedom from disease and decision-making for the cessation of control activities. We discuss our results in the context of recent emergency responses to rabies outbreaks, many of which have been on relatively isolated islands. While our results are focused on island dynamics for simplicity, we expect these insights to be transferable to contiguous landscapes once control measures have reduced incidence to relatively localized foci.

## 2 Materials and methods

### 2.1 The epidemiological model

We developed an epidemiological model of rabies transmission and spread which we used to evaluate different mass vaccination strategies. Our model was based on the biting and movement behaviour of infectious domestic dogs and was a spatially explicit, stochastic simulation using a simple density-independent branching process (see Table 2 for parameter values and Fig. S1 for parameter distributions). We assume that each infectious case causes *k* secondary cases (‘offspring’), drawn from a negative binomial distribution, with *R*_0_ as its mean, which we assume to be 1.2 (Fig. S1A). Each secondary case was assigned a generation interval selected from a gamma distribution representing an incubation period plus a period of infection prior to transmission, to determine when new infections were generated (Fig. S1B). Using a 1 km^2^ grid, we probabilistically allocated the locations of secondary cases. To capture the local movement of infected dogs, secondary cases were displaced from their direct epidemiological predecessors according to a negative binomial-distributed dispersal kernel with probability 1 − *p* (Fig. S1C). To capture human-mediated transport of dogs, exposed offspring were assigned to a randomly chosen grid cell with probability *p*. The branching process formulation does not account for any effects of depletion of the susceptible population as the incidence of infection increases. However, since rabies incidence is not estimated to exceed 3% per annum, depletion of the susceptible population is assumed to play a negligible role. Further details on the model are available in the Supplemental Material, which includes videos of model simulations.

### 2.2 Detection probabilities

**Fig. 1:**
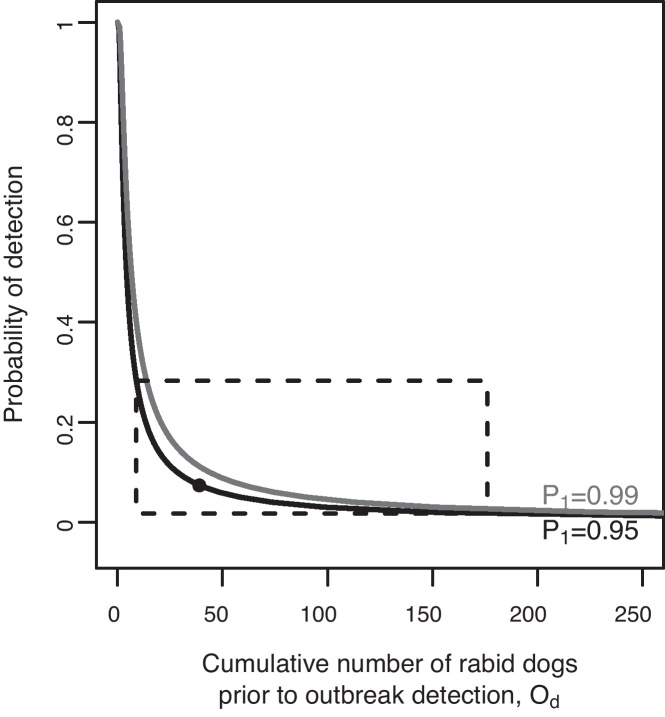
Probability of detecting a rabies outbreak. Solid lines indicate 0.99 (black) and 0.95 (grey) probabilities of detecting at least one case (*P*_1_). The black dot marks the median estimated probability of detection (*D* = 0.07) based on the median outbreak size (*O*_d_ = 39 cases) estimated from 10,000 model simulations of 7 months of rabies spread with no control, with *P*_1_ = 0.95. The dashed box indicates the 95% percentile interval (*D* = 0.02–0.28, *O*_d_ = 9–176 cases).

We conducted a literature search on rabies surveillance and outbreak detection and response times to recent canine rabies incursions (Web of Knowledge and PubMed for ‘rabies’ AND ‘outbreak’ OR ‘surveillance’ OR ‘incursion’ OR ‘response’ OR ‘containment’) and summarized features of these outbreaks and control operations (Table 1). We used both theory and empirical data to inform the detection probabilities explored in the model. Bacon [22] provides a relationship between outbreak size at the time of detection (*O*_d_) under different probabilities of detection (*D*): *D* = 1 − (1 − *P*_1_)(1/*O*_d_) where *P*_1_ is the probability of detecting at least one animal with rabies (Fig. 1). This relationship indicates that when the probability of detection exceeds 0.3, there are only negligible differences in outbreak size at the time of detection. Simulating an incursion following an index case for a period of 7 months until assumed outbreak detection (as likely occurred on Bali, Table 1) provides an estimate of probability of detection of around 0.07 (95% CI 0.02–0.28, Fig. 1). Based on these data, we considered detection probabilities in the range 0.01–0.30.

### 2.3 Model scenarios

**Fig. 2:**
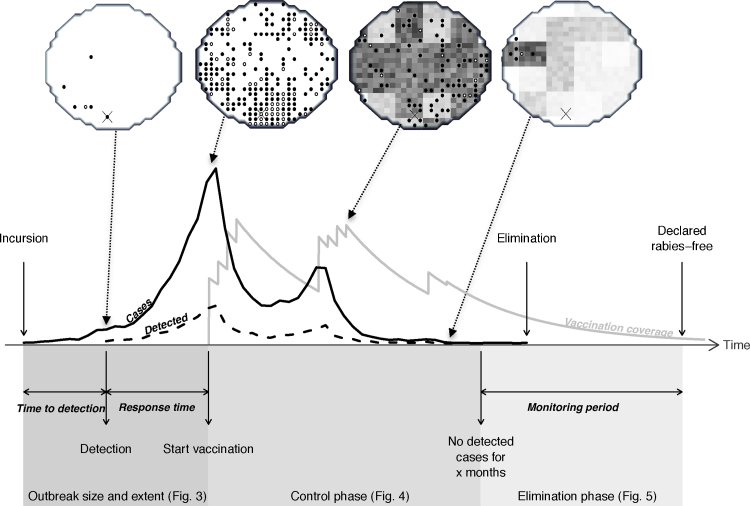
Simulation scenario indicating the critical time points from an incursion to the declaration of freedom from rabies. An example simulation illustrated as a time series and as the spatial occurrence of cases on an island grid (circular, 500 km^2^). During an outbreak, incidence (black solid line/dots indicates cell is infected) generally increases exponentially from the time of the incursion (cross marks incursion location). The delay to detection and therefore the number of detected cases (black dashed line/white dot indicates cell contains detected cases) varies according to the probability of detecting cases. Following outbreak detection, there may be a delay to implementation of a control strategy. Vaccination coverage (grey line/darker shading of cells indicates higher coverage) increases during campaigns and decays between campaigns due to waning of immunity and dog population turnover. This population would be considered rabies-free after a period of 2 years monitoring without any detected cases. Some undetected cases occur after the last detected case, but in this simulation the epidemic was extinct when freedom from rabies was declared. The model that generated this realization was used to generalise results from thousands of simulations, presented in Figs. 3–5.

We modelled surveillance as a probabilistic process sampling simulated rabies cases, with detected (i.e. sampled) cases used to trigger responses and determine decisions for subsequent interventions including the declaration of freedom from disease and the cessation of control activities (Fig. 2 illustrates an example of these time points). Specifically, we set up scenarios to explore the impact of the probability of detecting rabies cases during three phases: (1) from incursion to detection and mobilisation of a response, i.e. mass dog vaccination; (2) from the start of mass vaccination until control of the disease; and (3) from control to elimination. The three phases are highlighted in Fig. 2 with reference to the figures in which corresponding results are presented. Aspects of these phases were guided by data on recent rabies outbreaks where possible (Table 1, summarised in the bottom row). ‘Reference scenario’ refers to default model parameters and initialization. We initiated epidemics under a variety of scenarios characteristic of different populations (with differing island sizes and levels of human-mediated long distance transport of dogs) and environments (variable island shape that may hinder or facilitate disease spread) (detailed in Table 2). For each scenario explored, we ran 100 realizations in MATLAB (version 7 release 14, The MathWorks Inc.). An illustrated example of a model simulation of the reference scenario is shown in Fig. 2, and videos of simulations are available as Supplementary Data (Videos S1–S3).

For the first phase, incursion to mobilisation of a response, epidemics were seeded with a single randomly placed index case. The response time consisted of two periods: the time between incursion and outbreak detection, and a surveillance-independent period between detection and mobilisation of a response (0, 6 or 12 months).

For the control phase, we investigated which mass vaccination strategy was most effective under different initial conditions and levels of detection probability. To initialize conditions for this phase, we ran the model until a set number of infections had occurred (5000 for the reference scenario, Video S1 shows an example simulation). We then implemented vaccination (see Videos S2 and S3 for example simulations) and explored the effectiveness of responses in terms of: (a) the time to bring the outbreak under control, measured as the length of time for which the intervention needed to be maintained until 6 consecutive months passed with no detected cases; (b) the effort required to achieve control; and (c) the probability of elimination within 2 years of control following the suspension of vaccination campaigns.

Dog vaccination was represented in the model by reducing the number of secondary cases per primary infection in direct proportion to vaccination coverage at the time of transmission. The effects of rabies incidence on the proportion of dogs vaccinated were not incorporated as they were assumed to be negligible. We assigned vaccination coverage to each island grid cell (1 km^2^) which, during a campaign, was drawn from a uniform distribution between 35 and 70% to capture realistic variation in coverage achieved at the time of vaccination. We modelled waning of vaccination coverage according to dog demographic rates and the duration of vaccine-induced immunity (Table 2). Campaigns were implemented in the model over a 4-month period, once a year, with the total land mass divided into 32 blocks representing administrative areas. Therefore, each month, a coverage level was designated to grid cells from up to 8 blocks selected according to the response strategy. Responses (Table S1) were island-wide, whereby the whole island was vaccinated (‘proactive’, see Video S2 for a model simulation), or selectively conducted in blocks with detected infections. Within the category of reactive responses we included a strategy where blocks were not re-vaccinated during the same campaign (‘react-without-repeat’, see Video S3 for a model simulation). We also explored a proactive strategy whereby blocks were vaccinated in an order that prioritizes those with the most detected cases. We measured the relative effort required to implement each strategy by summing the number of blocks vaccinated to bring the outbreak under control, a measure that combines the number and extent of annual campaigns that resulted in control.

We considered the comprehensiveness of vaccination coverage on the prospects for elimination. Coverage was improved by achieving uniformly high (70%) coverage in every 1 km^2^ grid cell (‘hom’, in the reference scenario vaccination coverage is heterogeneous ‘het.’). We also modelled poorer coverage mimicking issues such as incomplete island-wide vaccination, coordination problems or inaccessible populations (‘patchy coverage’): for ∼20% of blocks (6 randomly chosen blocks), for each block we assigned vaccination coverage to each grid cell from a uniform distribution where the upper limit was a random number between 0 and 70% (e.g. 52%) and the lower limit was half the value (e.g. 26%). In further scenarios we investigated parameters that might affect the performance of reactive strategies, including a 14-day lag in the confirmation of cases, reactions based on several months of cases, and clustered epidemics.

For the elimination phase, we explored decisions necessary to determine freedom from disease given realistic probabilities of detection. Current guidelines state that rabies-free status is assigned following 2 years without cases, but do not indicate whether vaccination should continue during this monitoring period. We therefore explored the probability of elimination in the 2 years following a 2 or 6-month period without any detected cases, whereupon vaccination activities were halted, or continued for the 2-year duration. For simulations where rabies persisted because cases were not detected causing control to be stopped prematurely, we estimated the length of the monitoring period needed to ensure the detection of re-emergence.

## 3 Results

**Fig. 3:**
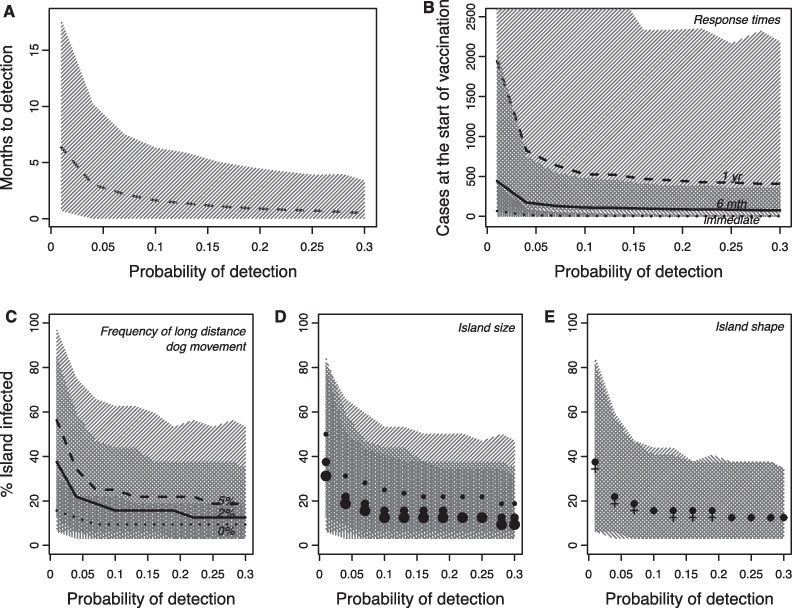
Outbreak size and extent under different detection probabilities. (A) Median interval between the index case and detection of the outbreak (dotted line). Shaded areas represent 95% CIs for all panels. (B) Outbreak size when a response is implemented: the reference scenario of 6 months to mobilization (‘6 mth’), as well as an ‘immediate’ and a slower response (‘1 yr’). (C–E) The extent of outbreaks (% blocks infected) at the time of detection under different detection probabilities and (C) long-distance (human-mediated) dog movement: 0%, 2% (reference) and 5%; (D) island sizes (large 15,000 km^2^, reference 5000 km^2^, small 500 km^2^); and (E) shapes: interdigitated islands (+) and circular islands (reference, **·**). Table 2 gives the model set up and parameters. These scenarios generate very different case distributions, potentially affecting the best vaccination strategy, which is considered in Fig. 4, Figs. S2 and S3.

The percentage of dog rabies cases detected in Bali was estimated at around 7% (95% CI: 2–28%, Fig. 1), and therefore we considered detection probabilities in the model across the range 0.01–0.30. The probability of detection affects the delay until an incursion is detected and therefore the epidemic situation can be markedly worse by the time that control efforts are initiated, with increasing outbreak size and extent at lower detection probabilities (Fig. 3A, B). For example, when the probability of detection is just 0.01, it could take 18 months for an outbreak to be detected (Fig. 3A) and, given a 6-month lag to initiate a response, almost 2000 dogs could become infected in the 2 years prior to implementation of control measures (Fig. 3B). Outbreak extent is further exacerbated in settings with more human-mediated long distance transport of dogs, on relatively smaller islands and, to a lesser degree, in areas with less complex coastlines/edges (Fig. 3C–E).

**Fig. 4:**
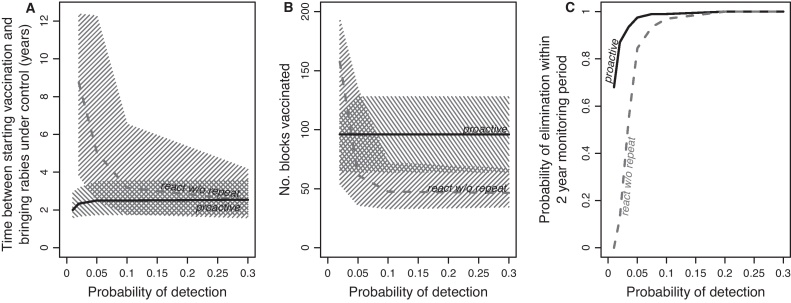
Impacts of the probability of detection on the effectiveness of mass vaccination strategies. (A) The time to control an outbreak (interval between starting vaccination and 6 months with no detected cases) under the proactive and react-without-repeat strategies. In A and B, median values are lines and hatched areas correspond to 95% CIs. (B) The effort required to control an outbreak measured as the number of blocks vaccinated. (C). The probability of elimination during the 2-year monitoring period after suspension of control efforts. See Table 2 for model set up and parameters, and Table S1 for vaccination strategy descriptions.

The probability of detection also affected the effectiveness of different mass vaccination strategies. Implementing the reference scenario model (Table 2) with proactive vaccination, the time to bring an epidemic under control (where successful) was consistently low (2.5 years, 95%CI: 1.5–3.5 years) across the range of detection probabilities (Fig. 4A) and lower with fewer starting cases (Fig. S2A). However, the response strategies that were dependent on the probability of detection showed greater variation in controlling rabies. The strategy which was proactive but prioritised the order of vaccination by the number of detected cases did reduce the time to control on average, but could increase the time to control when surveillance was poor (Fig. S2B). The react-without-repeat strategy was potentially as effective as proactive vaccination at bringing the epidemic under control, but often took considerably longer, especially at low detection probabilities (Fig. 4A) which negated any advantage of reduced effort of a reactive over a proactive strategy (Fig. 4B).

Once disease was brought under control following a 6-month period without any detected cases, the probability of achieving elimination in the 2-year monitoring period with mass vaccination suspended was high for all strategies at detection probabilities above ∼0.10 (Fig. 4C). However, at detection probabilities below 0.10 the probability of elimination was much lower for reactive vaccination than proactive vaccination, and declined to zero when detection probability was 0.01 (Fig. 4C). Under all the conditions that we explored (Fig. S3), the probability of elimination within the 2-year monitoring period was lower for reactive than for proactive vaccination.

Assuming comprehensive high coverage (in contrast to the heterogeneous coverage implemented under the reference scenario, ‘het.’ Fig. S2C), resulted in a greater chance of elimination (>95%) at almost all detection probabilities, only declining below 95% for very poor surveillance (0.01) as vaccination was suspended prematurely due to the substantial under-reporting (‘hom.’, Fig. S2C). Patches of low coverage profoundly damaged prospects of elimination for all probabilities of detection by creating pockets where rabies could persist (‘patchy coverage’, Fig. S2C).

**Fig. 5:**
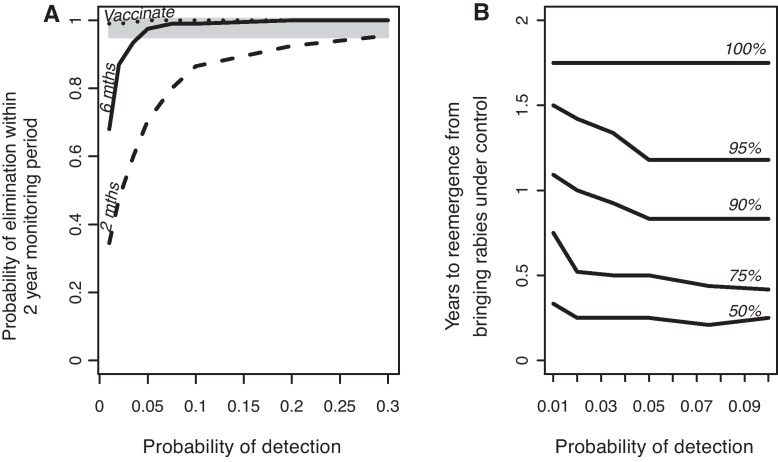
Prospects for elimination in relation to guidelines for suspending control activities. (A) The probability of elimination in a 2-year monitoring period following proactive vaccination until no cases were detected for 2 or 6 months. In the ‘vaccinate’ scenario, vaccination was continued during the 2-year monitoring period. The grey shading indicates a probability of elimination exceeding 0.95. (B) The time between stopping vaccination because the outbreak is perceived to be under control (6 months without any detected cases; solid line in A) and the detection of any re-emergence. Only probabilities of detection ≤0.1 were explored, as elimination was extremely likely at higher probabilities (solid line in A). This plot is based on 100 runs where rabies re-emerged, with confidence contours indicating the proportion of runs where rabies re-emerged within a given time period and at a given probability of detection.

Finally, we explored decisions necessary to determine freedom from disease. With the condition of no detected cases for a 6-month period being used to suspend mass vaccination, we found that the probability of elimination during the following 2-year period was very high (>0.99) for detection probabilities of at least 0.1 (Fig. 5A). However for lower detection probabilities the probability of achieving elimination rapidly declined, and became unacceptable (<0.95) at detection probabilities of less than 0.05 (Fig. 5A). Using a less stringent condition of 2 months with no detected cases to suspend control operations resulted in far lower probabilities of achieving elimination such that even with high levels of surveillance the probability of elimination was unacceptable (<0.95) (Fig. 5A). If vaccination campaigns were maintained throughout the 2-year monitoring period the probability of elimination was >0.95 for all detection probabilities (Fig. 5A). However, if vaccination efforts were suspended after 6 months with no cases, any re-emergences would still be detected within 1.75 years (21 months) of stopping vaccination under even the very poorest detection probabilities (Fig. 5B).

## 4 Discussion

Recent emergences of rabies and increasing momentum for rabies elimination programmes highlight the need for technical guidance and contingency planning to prevent outbreaks, respond to incursions should they occur and strategically implement control measures to meet elimination targets. Effective surveillance is integral to these objectives, however we have only a limited quantitative understanding of how surveillance quality might jeopardize these goals. The low incidence of rabies (<2.5% in Tanzania [23], and lower attack rates reported from elsewhere [10,11,24–27]) hinders the use of random sampling as a surveillance strategy for rabies. The large numbers of samples that would be needed to confirm a single rabies case would be both ethically unacceptable and logistically implausible. Applying WHO guidelines to sample 0.01–0.02% of the dog population would not generate sufficient positive samples to ensure surveillance quality even from highly endemic areas if sampling was not targeted, and could not be relied upon to certify freedom from disease. In contrast, the distinctive signs of rabies in animals are a sensitive means of identifying suspect rabies cases. Therefore targeted surveillance of high-risk animals (i.e. that are biting, behaving strangely, morbid or found dead) should be used to enhance disease detection and can be used to monitor surveillance capacity even when rabies has potentially been eliminated. We use this context of targeted sampling to investigate how the proportion of rabies cases that are detected (i.e. surveillance quality) affects rabies control measures, including responses to an outbreak and ultimately the elimination of disease.

Very little empirical data exists that can be used to assess rabies surveillance quality around the world. Here, we estimated that less than 10% of cases were confirmed in the recent outbreak in Bali. During an epidemic in Serengeti district (∼80,000 km^2^), Northern Tanzania where annual rabies incidence peaked at 2.5% [23], but was on average between ∼1–2% when control measures were in place, it was only possible to retrieve samples for laboratory confirmation from ∼5–10% of identified cases using exhaustive contact tracing. This was because the suspect animal could not be found or had already deteriorated once it had been found. Extrapolating similar levels of incidence to the Bali dog population (∼430,000 dogs), suggests that between 5,000–10,000 cases occurred during the epidemic peak in 2009–2010. Around 450 cases were confirmed over this 12-month period indicating a similar case detection probability of 0.045–0.09. These data suggest that generally detection probabilities for canine rabies are low (<0.1), and are consistent with, if not lower than, surveillance levels in Europe for wildlife rabies during highly successful oral vaccination programmes [22]. While surveillance infrastructure in Bali is considerably better than in Tanzania, government surveillance is less intensive than contact tracing. We therefore might expect considerably lower levels of surveillance (detection probability of <0.1) in areas with poorer infrastructure and without research capacity and this may be further compounded in malaria endemic areas by misdiagnosis [28]. Of those incursions described in Table 1, most were only detected after several months and rabies may have been circulating undetected for much longer, especially in areas with poorer surveillance infrastructure. Even with improved infrastructure, detection probabilities relying on laboratory confirmation and exceeding 0.3 would be difficult to achieve in most populations without considerably intensified effort.

Our key finding is that control programmes ought to be able to maintain surveillance levels that detect at least 5% of all cases to have realistic prospects of eliminating rabies, and that surveillance with detection probabilities of more than 0.1 would be ideal. Given that routine surveillance in much of sub-Saharan Africa probably detects far less that 5% of cases, increasing surveillance capacity must be considered an urgent priority. Field tests that could be easily applied could greatly increase the probability of detection in places with the weakest surveillance infrastructure. Indeed rapid field-testing became an important tool in the campaigns that successfully eradicated rinderpest [29]. Currently available rapid diagnostic field tests for rabies have a lower sensitivity relative to the gold standard FAT [30] and should not be used to guide the use of post-exposure prophylaxis. However greater use of these tests and the development of more reliable tests could be used to boost the probability of detection to the levels necessary to guide elimination efforts. Another effective means of improving detection probabilities would be through greater intersectoral communication. If health officers promptly notify veterinarians/animal health workers when bite patients report to a clinic, and the latter rapidly investigate these incidents, then a far higher proportion of cases would likely be detected. Recent efforts in Bali to improve coordination between sectors appear to have had such an impact, and evidence from contact tracing studies indicates that the vast majority of case histories can be traced by investigating incidents of biting animals.

In terms of vaccination strategies that are most effective, previous work showed that reactive vaccination can outperform proactive vaccination, eliminating rabies more rapidly particularly in areas with little human-mediated transport of dogs [31]. However, this appears only to hold true under very high levels of surveillance (probability of detection >0.3) because all affected areas are reactively vaccinated, whereas if surveillance is poor many areas supporting rabies transmission may be neglected if rabies is not detected. Assuming imperfect surveillance where only a small fraction of cases are observed is much more realistic and suggests that protecting populations where rabies has yet to be detected but are vulnerable is an important element in an effective strategy. Indeed reactive vaccination for rabies which is currently the norm in endemic countries that lack (or do not implement) national rabies control strategies [32] or have operational surveillance systems, would be very unlikely to have long-term impacts on reducing rabies incidence and would certainly not lead to elimination. On the basis of our findings we would not recommend reactive vaccination at all unless sufficiently high levels of surveillance are first deployed that effectively show the disease has been reduced to low levels in a few remaining foci. If such high levels of surveillance can be reached, then reactive vaccination (without repeats) may be worth considering because of its considerably reduced cost (Fig. 4B) and therefore may warrant further consideration in the context of the final stages of elimination programmes.

Vaccination campaigns are usually not conducted with equal efficacy across the target population. We therefore incorporated scenarios with more realistic heterogeneity in coverage. A reasonable level of heterogeneity (as modelled in the reference scenario) reduced the effectiveness of vaccination and had implications for surveillance, with poorer surveillance greatly reducing prospects of elimination. Of greater concern however, is the substantial impact of patchy coverage. Relatively comprehensive control programmes can be jeopardized if control operations are substantially weaker in just a small proportion of the overall area (Fig. S2C). Thus at the same time as boosting surveillance, a minimum control capacity ought to be required throughout an area under consideration for elimination.

The potentially long incubation period of rabies in dogs [33] makes ascertaining whether rabies has truly been eliminated relatively difficult, despite long periods of no detected cases. We modelled the incubation period and the infection period together as a gamma distribution, i.e. the general interval (the time between infection and becoming infectious) parameterised from data on rabid dogs in Tanzania [23]. From this distribution, in 95% of cases, the generation interval will be less than 2 months and in a further 4% of cases will be 2–3 months. The probability of a generation interval longer than 6 months is 0.01, and longer than 1 year is very small but not impossible (10^−9^). This long tail of the generation interval distribution is reflected in the confidence measures of disease elimination (Fig. 5B). For example, given a detection probability of 0.01 there is a 50% chance that, if rabies still persists, re-emergence will occur within 5 months of bringing rabies under control (defined here as 6 months with no detected cases), whereas to be certain that re-emergence will not occur requires monitoring for 1.75 years after successful control (Fig. 5B). How the generation interval is modelled, in particular whether an appropriately stochastic model is used, will influence estimates of the monitoring period needed to guarantee elimination and recommendations must take this uncertainty into account. Based on these analyses, 2 years 3 months without detecting any cases would be a sufficient criterion for rabies-freedom, even in areas with the poorest surveillance.

For programmes that aim for rabies elimination, the current 2-year guideline seems effective if control measures are maintained. If however control measures are discontinued, surveillance must ensure that at least 10% of cases are detected otherwise there is an unacceptable risk rabies will not go extinct (>0.05) within the 2-year monitoring period (Fig. 5). The potential to continue control efforts during the monitoring period to certify freedom from rabies contrasts to diseases such as foot-and-mouth disease and rinderpest. For these diseases, control activities need to be halted to ascertain disease-freedom using serosurveillance, whereas for rabies there would be no such opportunity costs of maintaining control measures.

Our findings from this modelling study have important practical implications that may be useful to guide policies for rabies containment and elimination. Overall we recommend minimum requirements for surveillance capacity including detection of at least 5% and preferably 10% of all cases. For programmes aiming for disease elimination, we would recommend a proactive strategy of mass vaccination continued for a 2-year period following 6 consecutive months without any detected cases. Mass vaccinations should ideally achieve uniformly high coverage, but the most important consideration is to ensure that no areas are left unvaccinated as patchy coverage could enable disease to persist in unvaccinated pockets. Should decisions be taken to prematurely discontinue control activities during the 2-year monitoring period, sufficient surveillance mechanisms must be in place to prevent potentially disastrous consequences. Further investigation on how to maintain freedom from disease in contiguous landscapes where neighbouring areas may act as a constant source of re-infection will be investigated more fully in future. However with an effective surveillance system operating, where medical and veterinary workers ally to achieve One Health, 2 years of continuous monitoring and vaccination should be sufficient to guarantee elimination of a controlled outbreak from an isolated area not subject to repeat introductions.

## Figures and Tables

**Table 1:** Examples of recent emergence or re-emergence of canine rabies, documenting what is known or estimated about the site and date of incursion, how long it took to implement a response and what type of intervention was implemented. d = days, w = weeks, m = months, y = years and NA refers to unknown information.

Location of outbreak	Epidemiological history prior to outbreak	Estimated date of incursion	Date of detection (time between incursion and detection)	Suspected source of incursion	Site of incursion	Response and date (time between detection and response)	Outcome	Time between sampling and FAT results	Sources
Central Java, Indonesia	No detected cases for at least 10 y	Aug–Sep 1985	Sep 1985	Dog/s transported from neighbouring endemic West Java	Wonogiri district, South East of Central Java	Mass vaccination, culling and movement control of dogs, cats and monkeys began ∼Nov/Dec 1985 (2–3 m)	Outbreak controlled, but few cases reported >1 y later	NA	Waltner-Toews et al. [24]
Terengganu, East Malaysia	Rabies eliminated in 1950s	NA	Dec 1995	Dog on fishing boat	Coastal villages	NA	NA	NA	Loke et al. [34]
Flores, East Nusa Tengarra, Indonesia	Naive group of islands	Sep 1997	Nov 1997 (2 m)	3 dogs on fishing boat from Butung (Buton) Island, Sulawesi	Larantuka, town on eastern tip	Culling began early 1998 (**<**4 m); vaccination began 2001 (>3 y)	Endemic	>14 d for dogs	Bingham [35]; Windiyaningsih et al. [11]; Scott-Orr et al. [36]
Maluku Islands, Indonesia	NA	NA	Aug 2003	Dogs imported for meat trade from other Indonesian islands; Sulawesi (A.A.G. Putra pers. comm.)	NA	NA	Endemic	NA	ProMED-mail [20]
3 neighbouring districts in Eastern Bhutan	Rabies eliminated in early 1990s	May 2005	May 2005 (<1 m)	Neighbouring endemic India	Gongza village, Toetsho sub district, Tanshiyangtse district	Vaccination began 2005 (**<**6 m); impounding began Mar 2006 (10 m); no culling as Bhuddist; no mass vaccination	Controlled by Nov 07	NA	Tenzin et al. [27]
Limpopo province, South Africa	No detected cases since 1981 but region endemic	Aug 05 earliest human case	Feb 06 (>6 m)	Southern Zimbabwe or Mozambique	Vhembe district, bordering southern Zimbabwe	Central point vaccination intensified in Feb 06 (<1 m). Infrastructure already in place, ∼40% coverage in preceding years	NA	NA	Cohen et al. [37]
Bhutan-Chhukha district	Rabies eliminated in early 1990s	Late 2007	Jan 2008 (1–3 m)	Neighbouring endemic India	Southern villages of Dala subdistrict	Culling began Mar 2008 (6 w); no mass vaccination	Controlled by Jul 2008	NA	Tenzin et al. [10]
Bali, Indonesia	Naive island	Apr 2008	Nov 2008 (7 m)	Dog on fishing boat from Sulawesi (N. Dibia, pers. comm.)	Ungasan village, Badung regency, southern peninsula	Localised control began Dec 08 (1 m); island-wide vaccination began Oct 2010 (2 y)	Continuing island-wide vaccination with considerable reduction in incidence	3 d for dogs (A.A.G. Putra pers. comm.)	Knobel and Hiby [38]; Putra et al. [39]; Susilawathi et al. [9]
Nias, Indonesia	Naive island	Medic official bitten Dec 2009	Medic official diagnosed with rabies Mar 2010 (>3 m)	NA	NA	Emergency dog vaccination and culling began Mar 2010 (**<**1 m); intermittent control measures to date but funds obtained and capacity being mobilized; planned mass vaccination in mid 2012 (2.5 y)	Endemic	NA	http://st284015.sitekno.com/article/30848/endemic-rabies-in-nias-island.html; http://www.civas.net/content/two-people-died-rabies-nias
Summary	Island and continental settings including areas where previously eliminated and without a rabies history		Time to detection <1–7 m	Human mediated transport by boat, possibly by vehicle on land, and perhaps local dog movement in Bhutan	Nearby islands or from neighbouring provinces/countries	Initial response <1–6 m; time to mass vaccination <1 m to 3 y (or still awaiting)	Variable: a few outbreaks controlled or eliminated, while others became endemic	3–14 d (very limited data)	

**Table 2:** Model scenarios and parameters values for rabies transmission processes, characteristics of the environment and dog population, surveillance and response. Reference scenario parameters and model set up are in bold.

	Parameter	Value	Source/Rationale
Transmission	Shape and scale of gamma distribution modelling generation interval	Shape **1.46** days; scale **16.1** days	Hampson et al. [23]
	Mean and dispersion parameter of negative binomial distribution modelling *R*_0_	Mean **1.20**; *k***1.33**	Hampson et al. [23]; Townsend et al. [31]

Environment and population	Area	500, **5000**, 15,000 km^2^	Ambon Maluku, Indonesia ∼775 km^2^; Bali, Indonesia ∼5600 km^2^; Bohol, Philippines ∼4100 km^2^; Nias, Indonesia ∼5100 km^2^; 3 districts in Eastern Bhutan ∼7000km^2^; Flores, Indonesia ∼14,300 km^2^
	Geometry	**Circular**, interdigitated	To compare a minimum edge effect versus a large edge effect
	Human-mediated long distance dog movement	0, **2**, 5%	Estimated for Bali, Indonesia [31]
	Local movement spatial kernel: mean and dispersion parameter (*k*) of negative binomial distribution	Mean **0.88** km^2^; *k***0.285**	Hampson et al. [23]
	Annual dog population turnover	50%	We assume that 50% of dogs vaccinated die one year later, and that the birth and death rates are equivalent

Detection and response	Probability of detection	0.01–0.3, **0.1**	See methods: *Detection probabilities*
	Response mobilization time	1, **6**, 12 months	Table 1
	Lag between detected case and laboratory confirmation	**0**, 14 days	Table 1
	Time period of cases used to determine reactive vaccination	**1**, 6 months	
	Duration of immunity provided by vaccine	**2** years	Most commercial vaccines provide 1–3 years of protection
	Vaccination coverage achieved at time and place of vaccination (*V*)	*V* = 70%, ***V***∼**uniform (35%, 70%),***V* for 80% of island ∼uniform(35%, 70%) and *V* for 20% of island ∼uniform(*X*/2,*X*) where *X*∼uniform(0,70%)	70% target vaccination coverage is recommended by WHO and empirically and theoretically supported [40]. Due to the difficulty of estimating coverage and dog population sizes, coverage achieved is expected to be spatially variable and may often fall below the 70% target.
	Vaccination strategy	**Proactive**, prioritise, react w/o repeat, reactive (see Table S1)	Builds on strategies explored in Townsend et al. [31]
	Cumulative number of cases when start vaccination	500, **5000**	5000 is the approx. cumulative number of cases on Bali when mass vaccination started, assuming 10% of cases were confirmed
	Length of monitoring period	**2** years	OIE and WHO criteria for rabies-free status requires 2 years without indigenously acquired infection [8,21]
	Months without any detected cases before starting 2-year monitoring period	2, **6** months	
	Intervention during monitoring period	**No vaccination**, proactive vaccination	
