# Supplementary figures and images for: Surveillance guidelines for disease elimination: A case study of canine rabies
Source: Comp Immunol Microbiol Infect Dis. 2013 May;36(3):249–61. doi: 10.1016/j.cimid.2012.10.008 (PMC3693035; doi:10.1016/j.cimid.2012.10.008)

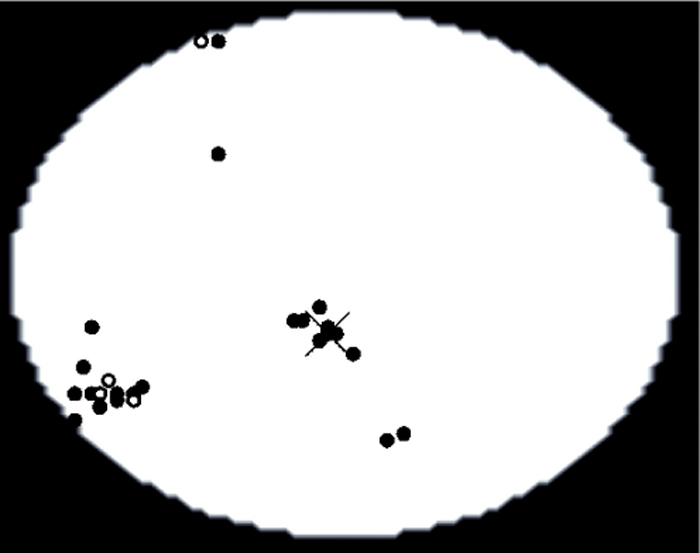

Supplement: Supplementary file 1 [file mmc1.jpg]

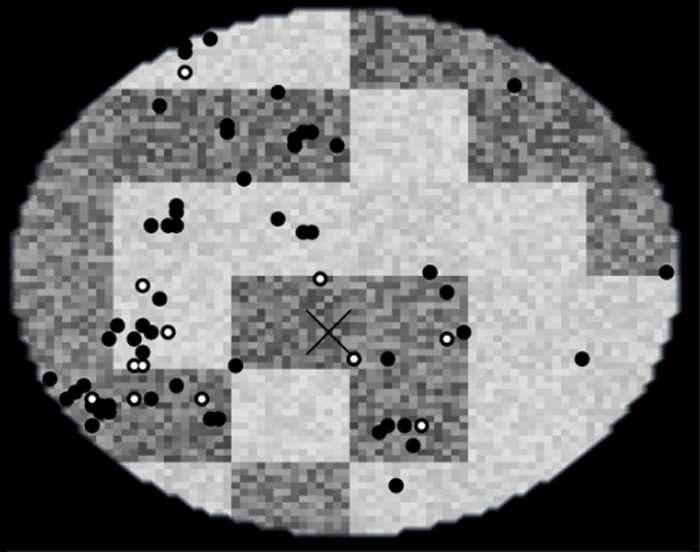

Supplement: Supplementary file 3 [file mmc3.jpg]

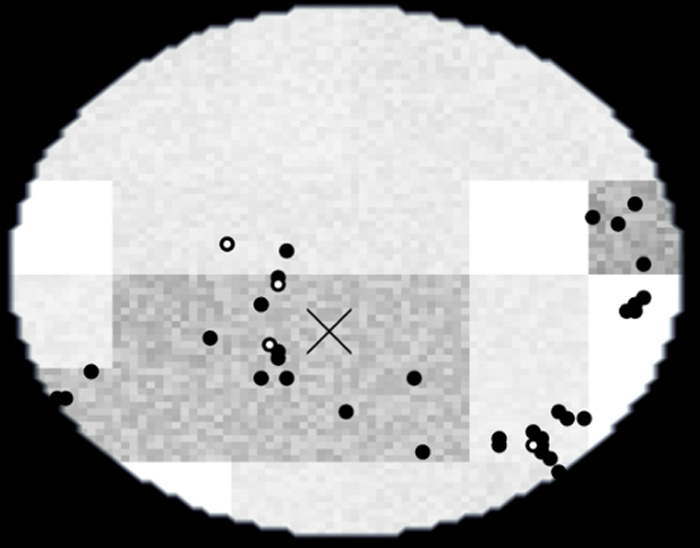

Supplement: Supplementary file 4 [file mmc4.jpg]
