# Supplementary material for: Surveillance guidelines for disease elimination: A case study of canine rabies
Source: Comp Immunol Microbiol Infect Dis. 2013 May;36(3):249–61. doi: 10.1016/j.cimid.2012.10.008 (PMC3693035; doi:10.1016/j.cimid.2012.10.008)
Supplement: Supplementary file 2 [file mmc2.docx]

**Supplementary Data**

To accompany: Townsend *et al.* 2012: Surveillance guidelines for disease elimination: a case study of canine rabies

**Further details on model methodology**

- We modelled vaccination coverage (the proportion of dogs vaccinated, *V*) in each cell as waning exponentially from the coverage achieved at the time of vaccination, at a rate (*Δt* = one day) determined by dog population turnover (*b* = birth rate and death rate, as population size appears roughly constant (M. Morters, *unpublished data*)) and the duration of the vaccine–induced immunity (*τ*, where v=1/*τ*):

$$V_{t+\Delta t}=V_{t}e^{-(v+b)\Delta t}$$

Parameter estimates are provided in Table 2.

- We made the conservative assumption that coverage did not accumulate over multiple vaccination campaigns i.e. we did not model an additive effect of vaccination coverage from previous campaigns i.e. if 70% of the population was vaccinated previously, but coverage dropped to 40% during the inter-campaign interval, a subsequent campaign reaching 70% of dogs only boosts coverage back to 70%. This is equivalent to assuming that previously vaccinated dogs are revaccinated in addition to vaccinating new dogs to bring coverage up to 70%.
- Dog vaccination is represented in the model by reducing the number of secondary cases per primary infection in direct proportion to vaccination coverage at the time of transmission. In effect, each potential secondary case becomes infectious with probability 1– *V_t_*, so in a vaccinated population the number of secondary cases attributed to each case is _v_~binomial(κ*,V_t_*).
- We assume no further introductions of rabies after the initial incursion.
- New infectious cases could only be allocated to a terrestrial grid cell.

| A | B | C |
| --- | --- | --- |
| **** | **** | **** |

**Fig. S1. Distributions used in the model of rabies spread** (A) Secondary cases are drawn from the offspring distribution, and (B) become infectious at a date drawn from the generation interval distribution. The occurrence of secondary cases depends on vaccination coverage in the grid cell at the time of transmission. (C) With probability 1–*p* each offspring occurs at a location generated from the local dispersal kernel. With probability *p*, each offspring occurs on any randomly chosen grid cell. See Table 2 for parameterisation of distributions.

**Table S1. Modelled vaccination strategies**, ordered by degree of reactivity to detected rabies incidence. All campaigns were annual and lasted 4 months. Islands were made of 1km^2^ grids divided into 32 blocks for vaccination. Fig. 2 and Video S3 show a simulation where the ‘react-without-repeat’ strategy was implemented. Video S2 is a simulation where the ‘proactive’ strategy was implemented. The effectiveness of the different strategies were measured in 3 ways: time to control an epidemic; effort required to achieve control and the probability of elimination within 2 years of control following the suspension of control efforts. Median (and 95% CI) effectiveness measures are given, assuming reference parameters which includes 0.1 probability of detecting cases (Table 2).

| Strategy | Description | Time to control of epidemic measured as years from response mobilisation to 6 months of no detected cases | Effort required to bring rabies under control measured as number of blocks vaccinated | Probability of elimination within 2 years of control with suspended vaccination |
| --- | --- | --- | --- | --- |
| Proactive | - Vaccinates blocks whether or not cases detected - Vaccinates blocks in random order - Order is the same for every round of vaccination over the course of a simulation - Does not re-vaccinate blocks in the same campaign - Surveillance quality has no impact on where vaccination takes place | 2.5 (1.8-3.5) | 96 (54-128) | 0.990 |
| Prioritise | - Vaccinates blocks whether or not cases detected - Vaccinates blocks with detected cases first, in order of incidence, then blocks without any detected cases in random order - Order is potentially different for every round of vaccination over the course of a simulation - Does not re-vaccinate blocks in the same campaign | 2.3 (1.6-4.4) | 96 (64-152) | 0.980 |
| React- without- repeat | - Only vaccinates blocks with detected cases (which depends on surveillance quality) - Does not re-vaccinate blocks in the same campaign - Order is potentially different for every round of vaccination over the course of a simulation | 3.2 (1.8-6.5) | 48 (33-72) | 0.970 |
| Reactive | - Only vaccinates blocks with detected cases (which depends on surveillance quality) - Always vaccinates blocks with the most detected cases regardless of whether vaccinated already during the campaign - Order is potentially different for every round of vaccination over the course of a simulation | 4.7 (2.7-9.5) | 126 (71-204) | 0.975 |

**Video S1 Simulation of a rabies outbreak of 5000 cumulative cases on a 5000km^2^ circular island.** The X marks the incursion location, solid black dots indicate cells that were infected in the last month, black dots with white centres indicate cells that contained detected cases in the last month. Parameters and model set up with the reference scenario (Table 2).

<http://ees.elsevier.com/cimid/download.aspx?id=12240&guid=6ea38d04-5037-4f91-bd73-152de8692c10&scheme=1>

**Video S2 Vaccination of the rabies outbreak in Video S1 with the proactive strategy**. Vaccination is represented by block colour, with darker colouring meaning higher coverage. Parameters and model set up with the reference scenario (Table 2).

<http://ees.elsevier.com/cimid/download.aspx?id=12242&guid=df2125d5-9ea7-4b3f-88ee-140a7c8321f2&scheme=1>

**Video S3 Vaccination of a rabies outbreak in Video S1 with the react-without-repeat strategy**. Except for vaccination strategy, parameters and model set up with the reference scenario (Table 2).

<http://ees.elsevier.com/cimid/download.aspx?id=12243&guid=0bcef65a-fd8b-4991-8efa-5f3191b962c7&scheme=1>

**Fig. S2. The performance of proactive vaccination under different conditions.** (A) The time to control the epidemic (where successful) under (A) different outbreak sizes: 500 or 5000 (reference) cases; (B) proactive vaccination in random order (reference) compared to a scenario where order is prioritised by the number of detected cases. Median times are lines and hatched areas are 95% CIs. See Table S1 for vaccination strategy descriptions. (C) The probability of elimination during the 2-year monitoring period after suspension of control activities once the outbreak is under perceived control (6 months with no detected cases) under variable vaccination coverage: comprehensive high coverage (‘hom.’), heterogeneous high coverage (‘het.’, reference) and high coverage with patches of low coverage (‘patchy coverage’). See Table 2 for model set up and parameters.

**Fig. S3. The performance of reactive vaccination under different conditions**: a 14 day lag between cases occurring and being confirmed when they inform the response (‘14 day lag’); vaccination order prioritized according to the number of detected cases occurring in the last 6 months (‘6 mths cases’); an epidemic that is highly clustered in its distribution (‘clustered’); an epidemic that is highly dispersed across the island (‘dispersed’).
